# Phylogenetic analysis of *Bacillus anthracis* strains from Western Siberia reveals a new genetic cluster in the global population of the species

**DOI:** 10.1186/s12864-019-6060-z

**Published:** 2019-09-02

**Authors:** Sergey V. Pisarenko, Eugene I. Eremenko, Alla G. Ryazanova, Dmitry A. Kovalev, Nina P. Buravtseva, Lyudmila Yu. Aksenova, Zorigma F. Dugarzhapova, Anna Yu. Evchenko, Elena V. Kravets, Olga V. Semenova, Olga V. Bobrisheva, Irina V. Kuznetsova, Tatyana M. Golovinskaya, Anna S. Volynkina, Sergei V. Balakhonov, Alexander N. Kulichenko

**Affiliations:** 1 Stavropol Research Anti-Plague Institute, 13-15 Sovetskaya Str, Stavropol, Russian Federation , 355035; 2 Irkutsk Antiplague Research Institute of Siberia and Far East, Irkutsk, Russian Federation , 664047

**Keywords:** *Bacillus anthracis*, Russia, Western Siberia, Whole genome sequencing (WGS), Whole-genome single-nucleotide-polymorphism analysis (wgSNP), Comparative genomics

## Abstract

**Background:**

Anthrax is a zoonotic disease caused by the gram-positive bacterium *Bacillus anthracis*. The most anthrax-endemic regions of Russia are Siberia and North Caucasus. Previously, genotyping of Russian *B.anthracis* isolates was carried out using canSNP and MLVA data; these methods yield lower resolution results compared to whole genome SNP analysis (wgSNP). In this research, we have used wgSNP method for genotyping of 10 *B.anthracis* isolates, obtained during 1961–2016 in Russia on territory of Western Siberia.

**Results:**

We have analyzed 185 *B.anthracis* genomes available in GenBank database and genomes of 10 isolates obtained in this study to determine the place of Russian isolates in the global phylogeny of *B.anthracis*. For the studied genomes we have detected 7203 SNPs, which were used for building a phylogenetic reconstruction with Maximum Likelihood Method. Results of the phylogenetic analysis indicate that Russian strains belong to three different genetic groups. Three strains belong to genetic group “Ames”, two strains – to “STI” group. Five strains belong to the main genetic line B, and four of them form a subcluster, described for the first time, which we have named “Siberia”.

**Conclusions:**

In this study, the data on genetic diversity of *B.anthracis* strains on the territory of Western Siberia is presented for the first time. As a result of complex phylogenetic analysis, the place of these isolates was determined in the global phylogenetic structure of the *B.anthracis* population. We describe a new cluster in the main genetic line B for the first time.

**Electronic supplementary material:**

The online version of this article (10.1186/s12864-019-6060-z) contains supplementary material, which is available to authorized users.

## Background

Anthrax is a particularly dangerous zoonotic disease caused by an aerobic gram-positive spore-forming microorganism *Bacillus anthracis*. Annually, between 2000 and 20,000 cases of anthrax are reported worldwide [1]. The anthrax pathogen infects livestock and wildlife, which inhale or ingest spores on pastures. Human infection usually occurs as a result of a contact with diseased animal or infected products and raw materials of animal origin. Depending on the mode of transmission (contact, inhalation, alimentary), cutaneous, respiratory or gastrointestinal forms occur. In their research Hanczaruk M. et al. describe another form, injectional, caused by injection of heroin, contaminated with anthrax spores [2]. The most endemic regions of the world are countries of sub-Sahara Africa, Central Asia, the Middle East and South America [3, 4]. In Russia, the greatest number of cases are registered on the territory of Siberia and the North Caucasus [5].

Western Siberia is a part of Siberia with an area of about 2451.1 thousand km^2^ (15% of the entire territory of Russia), located between the Ural Mountains in the west and the Yenisei River in the east. Anthrax epizooties emerged rather often on the territory of Western Siberia in the past. Mass summer mortality of deer was known centuries ago. This led to mass spore contamination of soil through discharge from diseased animals and their corpses. The largest in the past 37 years in Russia outbreak of anthrax occurred in 2016 in the north of Western Siberia in the Yamalo-Nenets Autonomous District. During this outbreak, 2649 diseased deer were found; 39 people were hospitalized, and 27 of them were diagnosed with anthrax via laboratory methods. There is very little published information on the strains of *B.anthracis* isolated in Russia. This information is limited to the description of the strain that caused the outbreak of anthrax in 1979 in Sverdlovsk and several strains, isolated in the tundra zone of Russia [6–10].

In this study we describe genetic diversity of *B.anthracis* strains isolated from different sources during anthrax outbreaks in Western Siberia from 1961 to 2016. In order to get a clear picture of the phylogenetic structure of the *B.anthracis* population in Western Siberia, we analyzed the strains using the whole genome SNP analysis (wgSNP). The obtained data on phylogenetic structure of Russian *B.anthracis* strains allowed us to complement significantly the current understanding of the phylogenetic structure of the global population of the anthrax pathogen.

## Results

### General results

**Fig. 1:**
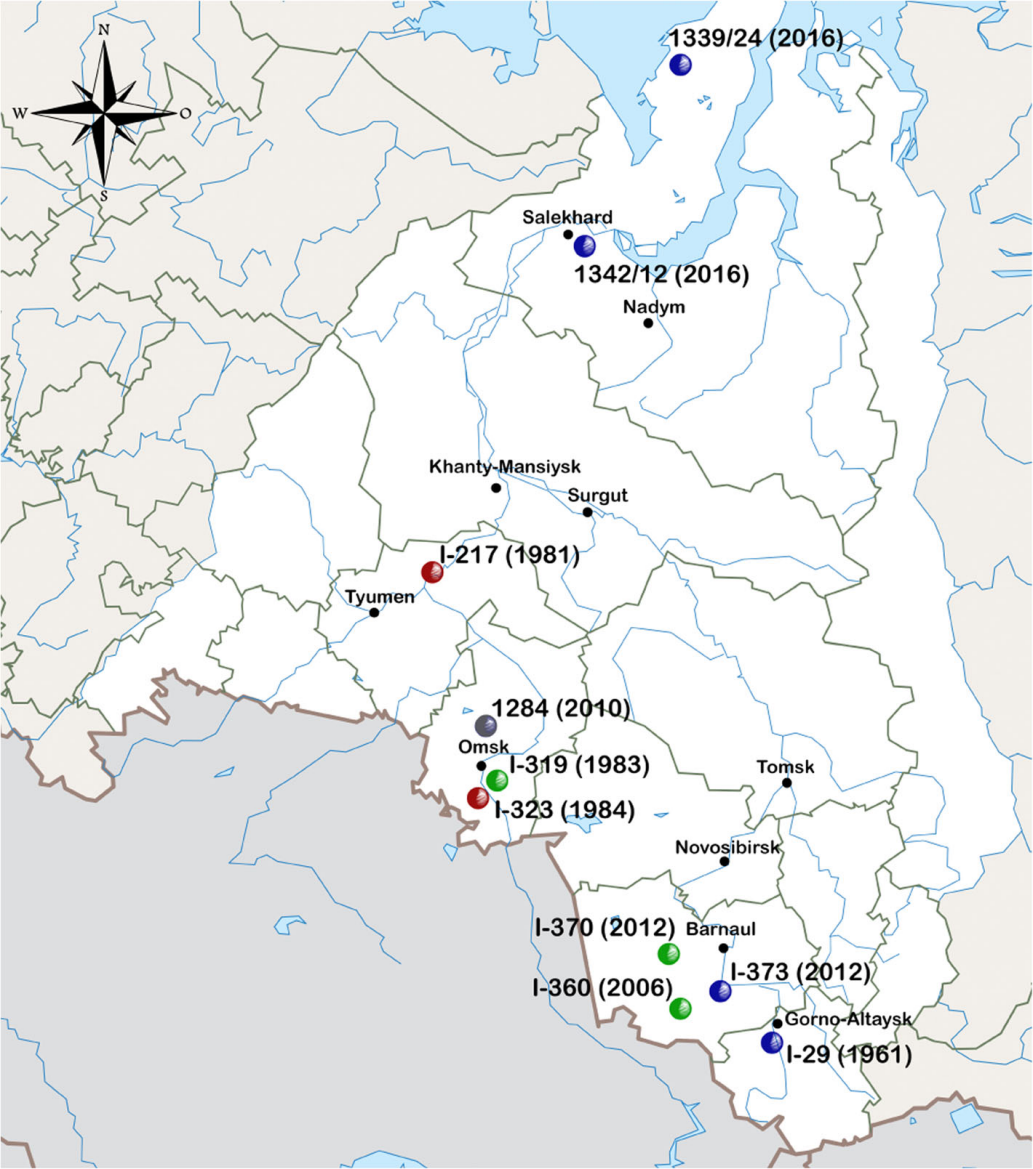
Overview map of Western Siberia. Markers on the map indicate the geographical location of the isolation sites of the strains in the respective regions of Western Siberia. The color of the marker indicates that the strain belongs to the genetic group, red - “STI”, green - “Ames”, blue - “Siberia”. Values in parentheses correspond to the isolation year of the isolates

For the research we have selected 10 isolates of *B.anthracis* obtained on the territory of Western Siberia during anthrax outbreaks registered from 1961 to 2016. When selecting, we were guided by their geographical origin and year of isolation (Table 1, Fig. 1). The presence of virulence plasmids pXO1 and pXO2 was confirmed by real-time PCR analysis. Genomic sequences of 10 studied strains were obtained by high-throughput sequencing using the Ion Torrent PGM platform (Life Technologies, USA). During the assembly of genomes, 41 to 71 contigs (> 500 bp) per genome were obtained. Detailed characteristics of genomic sequences are presented in Table 2.

**Table 1:** Metadata on *B.anthracis* strains from Western Siberia, used in this research

Strain name	Year of isolation	Region	Settlement	Origin of isolation
1284	Aug. 2010	Omsk Region	Omsk city	Meat products
1339/24	July 2016	Yamalo-Nenets autonomous district	in the Lake Pisieto area	Blood of deer
1342/12	July 2016	Yamalo-Nenets autonomous district	Salekhard city	Cutaneous ulcer lavage
I-29	Oct. 1961	Altai Republic	Cheposh village	Carbuncle contents
I-217	May 1981	Tyumen Region	Tobolsk city	Carbuncle contents
I-319	July 1983	Omsk Region	Omsk city	Cutaneous ulcer lavage
I-323	Jan. 1984	Omsk Region	Omsk city	Meat products
I-360	Aug. 2006	Altai Territory	Maralikha village	Cutaneous ulcer lavage
I-370	Sept. 2012	Altai Territory	Druzhba village	Soil from the place of slaughtering of bovine
I-373	Nov. 2012	Altai Territory	Bystryj Istok	Meat of a calf

**Table 2:** Characteristics of genomic projects

Strain	№ of Contigs	N50, bp	Total length, bp	GC, %	Genes (total)	CDS (total)	rRNAs	tRNAs
1284	52	237,795	5,437,248	35.09	5815	5730	1	84
1339/24	71	128,414	5,351,487	35.10	5806	5742	1	63
1342/12	47	274,537	5,432,938	35.09	5854	5778	1	75
I-29	41	630,126	5,433,410	35.09	5833	5757	1	75
I-217	69	227,313	5,373,867	35.12	5783	5698	1	84
I-319	50	239,741	5,447,981	35.09	5831	5747	1	83
I-323	42	237,851	5,172,041	35.22	5560	5484	1	75
I-360	54	202,591	5,443,985	35.09	5849	5773	1	75
I-370	65	157,503	5,443,288	35.09	5871	5795	1	75
I-373	51	270,707	5,433,661	35.09	5819	5734	1	84

### The place of *B.anthracis* strains isolated in Western Siberia in global population

The genomic sequences of 10 *B.anthracis* strains from Western Siberia, obtained in this study, and 185 complete *B.anthracis* genomes available in the international GenBank database were used to construct a global phylogeny of *B.anthracis* based on the SNP analysis of the complete genomes. Detailed information on genomes used in the analysis from the GenBank database is presented in Additional file 1: Table S1. Multiple genome alignment of 194 strains to the genome of the reference *B.anthracis* Ames Ancestor strain (as described in the Methods section) made it possible to detect 7203 SNPs, which allowed differentiation of the studied strains. These SNPs were used for phylogenetic reconstruction using the Maximum Likelihood method. A detailed description of all the SNPs used for phylogeny reconstruction is in Additional file 2: Table S2. Additional file 3: Figure S1 shows a phylogenetic tree describing the phylogenetic relationships of 195 strains of the *B.anthracis* species used in the analysis. The main genetic line C includes five strains of North American origin. Five Russian strains from Western Siberia were grouped in the clade B.Br.002. Moreover, four strains, 1339/24, 1342/12, I-29 and I-373 form a separate subclade in the clade structure B.Br.002. We have named this subclade “Siberia”. For these strains, we have detected 50 specific SNPs (see Additional file 2). We consider SNP to be specific if polymorphism was detected for every strain of “Siberia” subclade and was not present in any other strain, selected for the analysis. Strains of “Siberia” subclade are most closely related to HYU01 strain from South Korea and strains from “Kruger” subclade. Fifth strain, 1284, does not belong to “Siberia” subclade; instead, together with BA2968 strain from Finland, it makes a separate branch of B.Br.002 clade. The main genetic line A has a more complicated structure and includes 169 strains. There are many clusters, including major polytomy, called Trans-Eurasian (TEA) group. Two strains from Western Siberia, I-217 and I-323 belong to subclade “STI” of TEA 008/011 group. Apart from West-Siberian strains, this subclade includes five strains from Georgia, two isolates of vaccine strain STI and vaccine strain 55-VNIIVViM from Russia. Three other Russian strains, I-319, I-360, and I-370 belong to subclade “Ames” of A.Br.002 branch. Inside this subclade they are most closely related to Japanese strain Shikan-NIID.

## Discussion

Cluster structure of the phylogenetic tree built on the basis of wgSNP analysis correlates with previous descriptions based on lower resolution methods [11]. Phylogenetic analysis shows three main genetic lines A, B, C; moreover, lines B and C are less representative, than line A. For description of phylogeny, we used a previously described designation scheme.

**Fig. 2:**
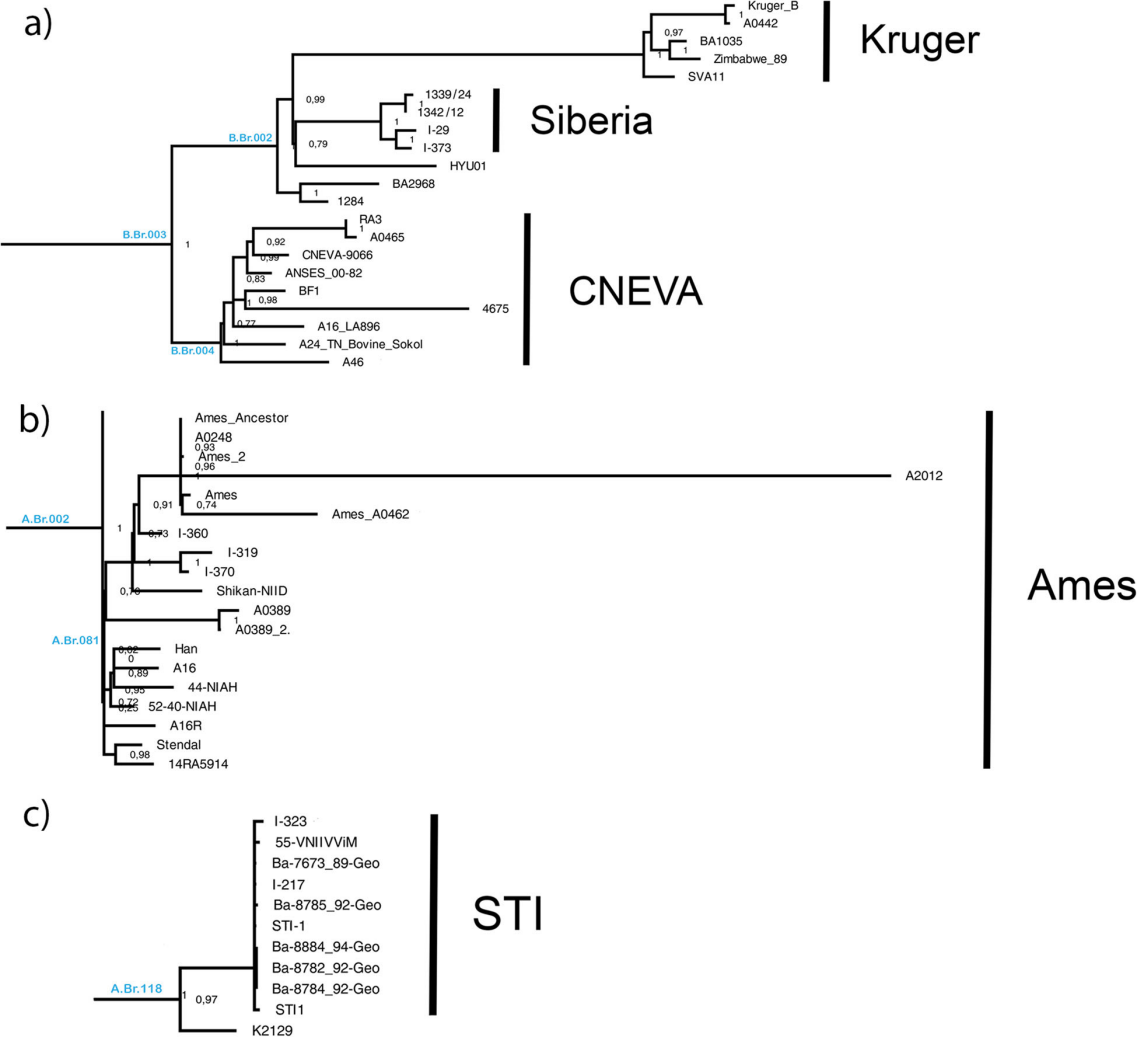
A close-up of fragments of a phylogenetic reconstruction based on wgSNP analysis of 195 *B.anthracis* genomes. **a** structure of A.Br.118 branch, **b** structure of A.Br.081 branch. **c** structure of B.Br.003 branch

Strains, isolated in Western Siberia from 1961 to 2016, belong to genetic lines A and B. Five out of ten Russian strains belong to B.Br.002 branch of main genetic line B (Fig. 2, Fragment a). Strains 1339/24 and 1342/12, isolated during an anthrax outbreak on Yamal in 2016, I-29 strain, isolated in the Republic of Altai in 1961, and I-373 strain, isolated in the Altai territory in 2012, form a separate subclade, which we have named “Siberia”. One must note that strains 1339/24 and 1342/12 were isolated in the northern part of Western Siberia, while strains I-29 and I-373 - in the southern. It seems likely that there was a common ancestor of these isolates, which had circulated throughout the entire territory of Western Siberia in the past. This hypothesis could have been proved by the data on strains that circulated in the period of 1950s and 1960s in central and northern parts of Western Siberia; unfortunately, isolates obtained during this period are not preserved to this day. Strains of “Siberia” subclade are most close to HYU01 strain, isolated from soil in South Korea in 2009 and the strains from South Africa, forming the “Kruger” subclade. The SNP analysis, performed through genome alignment to the reference *B. anthracis* Ames Ancestor strain, reveals from 791 to 797 nucleotide polymorphisms for strains of “Siberia” subclade (henceforward, referred to as SNPs are the ones found via multiple alignment method and filtered as described in Methods). Analysis of genetic markers that can help to differentiate strains of “Siberia” subclade from other *B.anthracis* strains revealed 50 strain-specific SNPs for “Siberia” subclade. Half of the detected specific SNPs are located in non-coding regions of the genome, the remaining 25 SNPs are located in the genes. A complete list of all specific SNPs with an indication of their localization regions is presented in Additional file 2: Table S2. The obtained information on specific SNPs can be further used to develop systems for the differentiation and intraspecific genotyping of strains of the anthrax bacterium.

*B. anthracis* strain 1284, isolated in the Omsk region in 2010, does not belong to “Siberia” subclade. According to the data of the anthrax outbreak investigation, this isolate was obtained from food products, derived from meat of animals infected with anthrax, which were imported into Omsk region from the territory of Kazakhstan without proper sanitary control. This fact fully explains the phylogenetic position of the *B.anthracis* strain 1284. *B. anthracis* strain BA2968 from Finland, isolated from a bull in 2008, is most closely related to *B. anthracis* strain 1284. SNP search through aligning to the reference *B. anthracis* Ames Ancestor strain detects 763 and 742 SNPs for strains BA2968 and 1284, respectively. This might be explained by the following: it is a known fact, that cases of anthrax in the countries of Northern Europe are extremely rare nowadays, due to anthrax programs implemented in the past [12, 13]. Lienemann T. et al. describe a possible route of *B.anthracis* strain BA2968 to Finland with imported products of animal origin [14]. To our mind, it is a quite logical and reasonable assumption. To prove this point of view, one might want to perform a phylogenetic analysis with the use of additional strains from Central Asia.

Main genetic line A has a complex structure, which includes a great number of subclades, as well as major polytomy, named Trans-Eurasian (TEA) group. Five West-Siberian strains, isolated from 1981 to 2012, belong to the main genetic line A. Two strains, I-360 and I-370, isolated in the Altay Territory in 2006 and 2012, respectively, and I-319 strain from the Omsk Region, isolated in 1983, belong to “Ames” subclade A.Br.002 branch (Fig. 2 Fragment b). SNP search compared to a reference *B.anthracis* Ames Ancestor strain detects from 39 to 62 nucleotide polymorphisms. These strains are closely related to Shikan-NIID, isolated from a horse in Japan in 1928. Strains that belong to this subclade are widely spread; they have been located in countries of Europe, Asia and America [15–17]. Taking into account topology of “Ames” subclade phylogeny, and the fact that Russian strains of this subclade have been isolated at different times during the last 38 years, one might assume that this genetic variant of the pathogen could have spread to the south territory of Western Siberia from Central Asia region. This spread could have been happening in the course of trading relations or in the course of military actions between the peoples, who inhabited these regions in the past millennium.

Two strains, I-217 and I-213, isolated in the Tyumen Region and Omsk Region in 1980s, belong to the “STI” subclade of the TEA group 008/011 (Fig. 2, Fragment c). For strains I-217 and I-323 we have detected 217 and 219 SNPs, respectively. Apart from two West-Siberian strains, to the “STI” subclade belong five strains, isolated in Georgia in 1989–1994, Russian vaccine strain 55-VNIIVViM and two isolates of the other Russian vaccine strain STI. Isolates STI-1 and STI1 are laboratory samples of an acapsular mutant STI-1, isolated from virulent strain of causative agent of anthrax “Krasnaya Niva” which became the basis of the Russian live anthrax vaccine STI for animals and people. Strains I-217 and I-323 do not group into a separate branch of a subclade; closest to them are vaccine strain 55-VNIIVViM and Georgian strains Ba-7673_89-Geo and Ba-8785_92-Geo. Until recent times, natural isolates that belong to the “STI” subclade have been detected only on the territory of Caucasus. However, Timofeev V. et al. in their research state that *B.anthracis* strain, isolated from permafrost in Yakutia, belongs to this subclade, too [10]. It is possible that genetic variants of the causative agent of anthrax which belong to the “STI” subclade are widely spread not only on the territory of Caucasus, but also on the entire territory of Siberia.

According to the results of the research, from 1961 to 2016 on the territory of Western Siberia *B.anthracis* strains which belong to three different genetic groups were isolated. Strains, isolated in the northern part of Western Siberia, in Yamalo-Nenets Autonomous Okrug, belong to the group “Siberia”. In the south, in the Altai Territory and the Altai Republic, at different times, strains of the two groups “Siberia” and “Ames” were isolated. Moreover, two strains I-370 and I-373 isolated in September and November 2012 in the Altai Territory, belong to different genetic groups. In the south-west, in Omsk and Tyumen regions, strains that belong to genetic groups “Ames” and “STI” were isolated at different times.

## Conclusion

Earlier it was reported that *B.anthracis* strains isolated in the tundra zone of Russia belong to the B.Br.001/002 branch of the main genetic line B [10]. The results of our study significantly expand the current understanding of the phylogeny of *B.anthracis* strains isolated in Russia. We have described genetic variety of *B.anthracis* strains, isolated on territory of Western Siberia in 1961–2016. Genotyping of the isolates was performed using the wgSNP analysis; this allowed us to determine their place in the global population of the causative agent of anthrax. The obtained data indicates that West-Siberian strains belong to three different genetic groups. We describe a new genetic group, “Siberia”, strains of which are spread in both northern and southern parts of Western Siberia. Further research of genomes of isolates of Russian *B.anthracis* strains will significantly complement the current understanding of the phylogenetic structure of the global *B.anthracis* population and will help expand the possibilities for differentiation of *B. anthracis* strains.

## Methods

### Bacterial strain

Ten *B.anthracis* strains used in this study were isolated from people, animals and natural environments on the territory of Western Siberia in 1961–2016. Strains 1284, 1339/24, and 1342/12 were obtained from the State Collection of Pathogenic Microorganisms of Stavropol Research Anti-Plague Institute, while the other 7 strains were transferred from the Irkutsk Anti-Plague Research Institute of Rospotrebnadzor in accordance with the Russian protocols “Procedure for organizing and conducting laboratory diagnostics of anthrax for laboratories at the territorial, regional and federal levels” (MUK 4.2.2941–11). In both institutes, the strains were stored in a lyophilized state. Metadata on these strains can be found in Table 1. Strains were identified using standard biochemical methods in accordance with the requirements of the MUK 4.2.2941–11. Antimicrobial susceptibility testing was performed using disc diffusion method with “Set of discs for antimicrobial susceptibility testing -1” (Institute Pasteur in Saint-Petersburg for Research in Epidemiology and Microbiology of Federal Service for Surveillance on Consumer Rights Protection and Human Wellbeing) in accordance with manufacturer’s instructions. Biochemical properties of the isolates are described in Additional file 4: Table S3.

### Diagnostic real-time PCR for chromosomal and plasmid markers of *B. anthracis*

For identification of *B. anthracis* by real-time PCR, we used the “Reagent kit for detecting *Bacillus anthracis* DNA in biological material and environmental objects by the method of polymerase chain reaction (PCR) with real-time hybridization-fluorescence detection” FRT” (Interlabservice, Russia) with amplification of specific genetic markers pagA (pXO1) and capA (pXO2). Real-time PCR analyses were performed using Rotor-Gene Q (QIAGEN, Germany).

### Growth of *B. anthracis* and extraction of DNA

Vegetative cells of *B.anthracis* strains were cultured on blood agar, then inactivated, and sterile DNA was isolated using a DNA isolation kit QIAamp DNA Mini Kit (Qiagen, Germany) in accordance with the manufacturer’s protocol. All manipulations were performed in the biosafety laboratory level 3. Sterility of the DNA samples was confirmed by cultivating 5% of the final DNA volume with negative results. The DNA concentration was quantified using the Qubit dsDNA HS assay kit (Thermo Fisher Scientific, USA) according to the manufacturer’s protocol. DNA preparations were stored at − 20 °C until further use.

### DNA library preparation and whole genome sequencing

The preparation of genomic libraries with a 400 bp read length was performed using the Ion Xpress Plus Fragment Library Kit reagent kit (Life Technologies, USA) in accordance with the manufacturer’s protocol. DNA library fragments were separated using 2% E-Gel SizeSelect agarose gel (Invitrogen, USA). The finished libraries of DNA fragments were purified using Agencourt AMPure XP magnetic particles (Beckman Coulter, USA). Libraries quality and concentration were determined using the Experion™ Automated Electrophoresis System and Experion DNA 1 K Reagents and Supplies and Experion DNA Chips kits (Bio-Rad, USA). Monoclonal amplification on microspheres was performed using Ion PGM Hi-Q View OT2 Kit reagents (Life Technologies, USA) in accordance with the manufacturer’s protocol. Microsphere enrichment was performed using Dynabeads MyOne Streptavidin C1 magnetic particles (Invitrogen, Life Technologies, USA). The effectiveness of the enrichment process was evaluated using the Ion Sphere Quality Control Kit (Life Technologies, USA). Genome sequencing was performed using an Ion Torrent PGM sequencer and Ion 318 Chips Kit V2 chips (Life Technologies, USA).

### Post-sequencing data processing

The quality assessment of the obtained reads was performed using the FastQC version 0.11.3 program [18]. Reads with an average value of quality Q < 20, as well as reads with length of less than 50 nucleotides were removed in the Trimmomatic version 0.33 program [19]. The genomes were assembled using the Newbler v3.0 software (Roche, Switzerland). Assessment of the quality of genome assemblies was performed using the program Quast 5.0 [20], the genomic sequence of *B.anthracis* Ames Ancestor strain (GenBank: NC_007530.2, NC_007322.2, NC_007323.3) was used as a reference to assess the accuracy and efficiency of the genomic project assembly. Genomes were annotated using the program PROKKA [21].

### Whole genome SNP analysis

We used the Parsnp tool from the Harvest Suite software for fast multiple alignment of genomic sequences [22]. As input, we used the genomes of ten strains sequenced by us and the genomic sequences of 185 *B.anthracis* strains from the GenBank public database which were aligned against the chromosomal nucleotide sequence of the reference genome of *B.anthracis* Ames Ancestor (GenBank: NC_007530.2) using Parsnp (parameters -c -e -u -C 1000). The detected SNPs were extracted to a VCF file, using HarvestTools (version 1.0) from the same software package. To improve the overall quality of the data, SNP positions with a distance of less than 10 bp, as well as positions that carry an unspecified nucleotide (“N”) were removed. The edited file was used as an input file in HarvestTools to compile the FASTA file. Phylogenetic reconstruction was built in Mega10 [23] using the Maximum Likelihood method according to the Tamura-Nei model [24]. The bootstrap confidence values were generated using 1000 permutations. Visualization of the phylogenetic tree was performed in the Figtree program [25].

## Additional files

Additional file 1: **Table S1.** Strain metadata. (XLSX 23 kb)

Additional file 2: **Table S2.** Base of sites of nucleotide polymorphisms of 195 *Bacillus* strains. (XLSX 9461 kb)

Additional file 3: **Figure S1.** Phylogenetic clustering based on wgSNP analysis from 184 *B. anthracis* strains. (TIF 1938 kb)

Additional file 4: **Table S3.** Biochemical properties of strains. (DOCX 13 kb)

## Data Availability

Generated genomic sequences of this study are available from the Russian Federal Service for Surveillance on Consumer Rights Protection and Human Wellbeing (Rospotrebnadzor) but restrictions apply to the availability of these data, which were used under license for the current study, and so are not publicly available. Data are however available from the authors upon reasonable request and with permission of the Russian Federal Service for Surveillance on Consumer Rights Protection and Human Wellbeing (Rospotrebnadzor). Other sequences used for this study are publicly available in the National Center for Biotechnology Information in the repository under the following accession numbers: GCA_000585275.1, GCA_000732465.1, GCA_000697555.2, GCA_000697515.2, GCA_900013465.1, GCA_900014335.1, GCA_900014345.1, GCA_900014355.1, GCA_900012555.1, GCA_900014365.1, GCA_900013475.1, GCA_900014375.1, GCA_900013485.1, GCA_900011745.1, GCA_000697535.2, GCA_000808075.1, GCA_000830095.1, GCA_000512835.2, GCA_000512775.2 GCA_003063965.1, GCA_003063945.1, GCA_001277955.1, GCA_001543225.1, GCA_001654475.1, GCA_001936375.1, GCA_000831505.1, GCA_000295695.1, GCA_000292565.1, GCA_003063985.1, GCA_003064015.1, GCA_003064045.1, GCA_003064085.1, GCA_003063925.1, GCA_002896575.1, GCA_002896585.1, GCA_002896655.1, GCA_002896695.1, GCA_002896665.1, GCA_002896595.1, GCA_002896635.1, GCA_003064005.1, GCA_000740925.2, GCA_000559005.1, GCA_000558965.1, GCA_000558985.1, GCA_001277085.1, GCA_001276995.1, GCA_000986915.1, GCA_000986935.1, GCA_000875715.1, GCA_001273045.1, GCA_001273105.1, GCA_001272985.1, GCA_001273005.1, GCA_001273025.1, GCA_001273085.1, GCA_001273065.1, GCA_001273125.1, GCA_001273145.1, GCA_003368005.1, GCA_003367985.1, GCA_003345105.1, GCA_003045745.1, GCA_000008505.1, GCA_000008165.1, GCA_002277915.1, GCA_003335125.1, GCA_000782875.1, GCA_000793525.1, GCA_000782885.1, GCA_000782895.1, GCA_000782955.1, GCA_000793565.1, GCA_000782975.1, GCA_000782995.1, GCA_000783015.1, GCA_000783035.1, GCA_000783055.1, GCA_000783075.1, GCA_000793545.1, GCA_000783095.1, GCA_000782905.1, GCA_000783115.1, GCA_000783135.1, GCA_000783155.1, GCA_000783165.1, GCA_000783195.1, GCA_000783215.1, GCA_000783235.1, GCA_000725325.1, GCA_000521345.1, GCA_000022865.1, GCA_000167255.1, GCA_000006155.2, GCA_000007845.1, GCA_000008445.1, GCA_000167335.1, GCA_000021445.1, GCA_000167235.1, GCA_000167295.1, GCA_000167275.1, GCA_000167315.1, GCA_000181675.2, GCA_000258885.1, GCA_000742655.1, GCA_000832965.1, GCA_000743825.1, GCA_000833065.1, GCA_000832665.1, GCA_000832725.1, GCA_000742875.1, GCA_000833125.1, GCA_000742695.1, GCA_000832465.1, GCA_000832505.1, GCA_000832425.1, GCA_000832585.1, GCA_000832745.1, GCA_000832565.1, GCA_000742315.1, GCA_000832635.1, GCA_000833275.1, GCA_000832785.1, GCA_000832445.1, GCA_000742895.1, GCA_000743805.1, GCA_000182055.1, GCA_000181915.1, GCA_000219895.1, GCA_000181935.1, GCA_000181995.1 GCA_000181835.1, GCA_000310045.1, GCA_000007825.1, GCA_002025435.1, GCA_002025375.1, GCA_002025335.1, GCA_002025455.1, GCA_002025395.1, GCA_002025275.1, GCA_002025265.1, GCA_002019425.1, GCA_002025345.1, GCA_002025415.1, GCA_001015005.1, GCA_001015025.1, GCA_002356575.1, GCA_000583105.1, GCA_001835485.1, GCA_002005265.1, GCA_000319695.1, GCA_000319715.1, GCA_000278385.1, GCA_003227955.1, GCA_002980615.1, GCA_000534935.2, GCA_001029875.1, GCA_000747335.1, GCA_000747375.1, GCA_001883895.1, GCA_003410355.1, GCA_003410255.1, GCA_001883885.1, GCA_001758315.1, GCA_002559375.1, GCA_002558905.1, GCA_002558675.1, GCA_002558195.1, GCA_002561555.1, GCA_002561395.1, GCA_002561195.1, GCA_002561845.1, GCA_002561055.1, GCA_002563115.1, GCA_002560675.1, GCA_002560535.1, GCA_002560185.1, GCA_002559785.1, GCA_002559755.1, GCA_002559605.1, GCA_002583575.1, GCA_002584885.1, GCA_002584475.1, GCA_002584485.1, GCA_002585175.1, GCA_002577475.1, GCA_002577275.1, GCA_002576295.1, GCA_002576945.1, GCA_002575885.1, GCA_002574215.1, GCA_002574185.1, GCA_002574755.1, GCA_002571055.1, GCA_002570575.1, GCA_002570775.1, GCA_002567755.1, GCA_002570225.1, GCA_002567055.1, GCA_002564895.1, GCA_002566425.1, GCA_002566085.1, GCA_002553265.1, GCA_002552535.1, GCA_002550475.1, GCA_002550395.1, GCA_002007035.1, GCA_001990245.1, GCA_002024565.1, GCA_001683095.1, GCA_001683065.1, GCA_001683135.1, GCA_001683155.1, GCA_001683175.1, GCA_001683195.1, GCA_001683215.1, GCA_001683235.1, GCA_001683255.1, GCA_001683275.1, GCA_001683295.1, GCA_001677305.1, GCA_001677295.1, GCA_002233635.1, GCA_000359465.1, GCA_000359425.1, GCA_000359445.1, GCA_002208785.2, GCA_002525715.1, GCA_002525705.1, GCA_002525685.1, GCA_002525785.1, GCA_002525695.1, GCA_002525765.1, GCA_002525775.1.

## Abbreviations

SNP: Single Nucleotide Polymorphism
TEA: Trans Eurasion Group
WGS: Whole Genome Sequencing
wgSNP: Whole-Genome Single-Nucleotide-Polymorphism
