# Supplementary material for: Phylogenetic analysis of Bacillus anthracis strains from Western Siberia reveals a new genetic cluster in the global population of the species
Source: BMC Genomics. 2019 Sep 2;20:692. doi: 10.1186/s12864-019-6060-z (PMC6720099; doi:10.1186/s12864-019-6060-z)
Supplement: Supplementary file 4 — Table S3. Biochemical properties of strains. (DOCX 13 kb) [file 12864_2019_6060_MOESM4_ESM.docx]

Table S4. Biochemical properties of the isolates.

| Strain | pXO1 | pXO2 | Capsule | Phosphatase activity | Hemolytic activity | Lecithin activity | Gamma phage lysis | Penicillin sensitivity |
| --- | --- | --- | --- | --- | --- | --- | --- | --- |
| *B. anthracis* 1284 | + | + | + | - | - | - | + | + |
| *B. anthracis* 1339/24 | + | + | + | - | - | - | + | + |
| *B. anthracis* 1342/12 | + | + | + | - | - | - | + | + |
| *B. anthracis* I-29 | + | + | + | - | - | - | + | + |
| *B. anthracis* I-217 | + | + | + | - | - | - | + | + |
| *B. anthracis* I-319 | + | + | + | - | - | - | + | + |
| *B. anthracis* I-323 | - | - | - | - | ± | ± | + | + |
| *B. anthracis* I-360 | + | + | + | - | + | - | - | + |
| *B. anthracis* I-370 | - | - | - | - | - | - | + | + |
| *B. anthracis* I-373 | + | + | + | + | - | - | + | + |

«+» - positive, «-» - absent, «±» - partly
